# A prevalence survey of every-day activities in pregnancy

**DOI:** 10.1186/1471-2393-10-41

**Published:** 2010-08-04

**Authors:** Samantha J Lain, Jane B Ford, Ruth M Hadfield, Christine L Roberts

**Affiliations:** 1 Perinatal Research, Kolling Institute of Medical Research, University of Sydney, St Leonards, NSW, Australia; 2 Department of Obstetrics and Gynaecology, Royal North Shore Hospital, St Leonards, NSW, Australia

## Abstract

**Background:**

Research into the effects of common activities during pregnancy is sparse and often contradictory. To examine whether common activities are an acute trigger of pregnancy complications the prevalence of these activities are necessary to determine sample size estimates. The aim of this study is to ascertain the prevalence of selected activities in any seven day period during pregnancy.

**Methods:**

The study was conducted in the antenatal clinic of a teaching hospital with tertiary obstetric and neonatal care in Sydney, Australia between August 2008 and April 2009. Women who were at least 20 weeks pregnant and able to read English completed a questionnaire to assess whether they had performed a list of activities in the seven days prior to survey completion. Results were analysed using frequency tabulations, contingency table analyses and chi square tests.

**Results:**

A total of 766 surveys were completed, 29 surveys were excluded as the women completing them were less than 20 weeks pregnant, while 161 women completed the survey more than once. Ninety seven per cent of women completed the survey when approached for the first time, while 87% completed the survey when approached a subsequent time. In the week prior to completing the survey 82.6% of women had consumed a caffeinated beverage, 42.1% had had sexual intercourse, 32.7% had lifted something over 12 kilograms, 21.4% had consumed alcohol and 6.4% had performed vigorous exercise. The weekly prevalence of heavy lifting was higher for multiparous women compared to nulliparous women.

**Conclusions:**

The results of this study can be used to inform future research into activities as acute triggers of pregnancy complications.

## Background

Women have many concerns about the safety of performing otherwise everyday activities during pregnancy. Pregnant women seek information about air travel, sexual intercourse and exercise during pregnancy[1], while antenatal care providers may also need to confirm or refute pregnancy 'old wives' tales', such as eating spicy food to induce labour[2]. However research into the effects of these and other common activities during pregnancy is sparse and often contradictory[3-10].

To perform high quality research into the effects of these activities in pregnant women, each study needs a sample size large enough to demonstrate differences between groups of women. To calculate the sample size for a case-control or case-crossover study requires the prevalence of these activities during pregnancy. As most activities are transient in nature, the timing of these activities may be important as a possible acute trigger of pregnancy complications such as preterm birth, placental abruption, premature spontaneous rupture of membranes and preeclampsia. Pregnant women have been asked to report the frequency of performing every-day activities during specific trimesters[4,9,11-13] or any time during pregnancy[3,14-16]; however, to investigate these activities as acute triggers it is important to know their prevalence in a smaller window of time. The aim of this study is to ascertain the prevalence of selected activities in any seven day period during pregnancy.

## Methods

The study was conducted in the antenatal clinic of a teaching hospital with tertiary obstetric and neonatal care in Sydney, Australia between August 2008 and April 2009. Women who were at least 20 weeks pregnant and were able to complete the questionnaire in English were eligible. As the questionnaire sought information about events in the seven days prior to its completion, women were eligible to complete the survey more than once, providing there was at least fourteen days between each questionnaire.

Eligible women were approached by a researcher in the antenatal clinic waiting area and given written and verbal information about the study. The researcher was present in the antenatal clinic from Monday to Friday ensuring women receiving care from different health providers were recruited (49% of women were visiting General Practitioners, 40% were visiting a midwife and 11% were in a high-risk clinic). Women who consented to be in the study completed the survey while waiting for their antenatal appointment. The questionnaire was developed from a review of literature and discussion with clinical staff of possible acute triggers for conditions in pregnancy. Questionnaire development included pilot testing on fifteen women attending antenatal appointments and refinement. Minimal changes were required to the survey following pilot testing so it was decided that re-piloting was not necessary. The questionnaire took 3 to 5 minutes to complete and asked women about demographic characteristics and whether they had engaged in a list of activities in the seven days prior to completing the questionnaire.

Survey data were analysed using frequency tabulations and contingency table analyses using SAS, version 9.1 (SAS Institute, Cary NC, USA). Stratified analysis, using chi square tests, examined the impact of gestational age (20-28 weeks, 29-34 weeks, and 35 weeks or more), parity (first pregnancy compared to second or subsequent pregnancy), maternal age (less than 25 years, 25 to 34 years, and 35 years or more) and maternal education (university degree compared to no university degree) on prevalence of activities. Stratified analyses were performed based on number of women who completed the survey compared to total number of surveys to examine the effect of clustering of activities amongst women who completed the survey more than once. This study was approved by the Northern Sydney and Central Coast Human Research Ethics Committee.

## Results

Ninety seven per cent of the women approached the first time to participate in the study consented. When women were approached to complete the survey for a subsequent time 87% consented. A total of 766 surveys were completed, 29 surveys were excluded as the women completing them were less than 20 weeks pregnant, while 161 women completed the survey more than once. The majority of the women that completed the survey were over 30 years of age, had a University degree, and were having their first baby (Table 1).

**Table 1:** Distribution of demographic characteristics in the study population

	Women who completed survey for first time N = 576	Women who completed survey second or subsequent time N = 161
**Age (years)**	**N (%)**	**N (%)**
< 25	45 (7.9)	10 (6.3)
25 - 29	119 (20.8)	24 (15.0)
30 - 34	221 (38.6)	65 (40.6)
≥ 35	187 (32.7)	61 (38.1)
**Gestation (weeks)**		
20 - 28	217 (37.7)	24 (14.9)
29 - 34	134 (23.3)	38 (23.6)
35 - 40	225 (39.2)	99 (61.4)
**Plurality**		
Singleton	556 (96.7)	149 (92.5)
Twins/Triplets	19 (3.3)	12 (7.5)
**Number of previous pregnancies**		
0	294 (51.1)	82 (50.9)
1 or more	281 (48.9)	79 (49.1)
**Highest level of education completed**		
Secondary school or below	73 (12.7)	14 (8.7)
TAFE/Diploma/Certificate	183 (31.9)	83 (26.7)
University degree	318 (55.4)	104 (64.6)
**Medical conditions (pre-existing or pregnancy related)**		
Asthma	50 (8.7)	18 (11.2)
High blood pressure	21 (3.6)	6 (3.7)
Diabetes	37 (6.4)	14 (8.7)
Other	24 (4.2)	7 (4.4)
**Smoking status**		
Smoked cigarettes prior to being pregnant	85 (14.8)	26 (16.2)
Smoked cigarettes during pregnancy	24 (4.2)	5 (3.1)

The weekly prevalence rates for recent activities, calculated from all surveys completed, are outlined in Table 2. The most prevalent activity in the week prior to completing the survey was drinking caffeinated beverages, this included coffee, tea or cola drinks. The activities occurring most infrequently were taking medication for depression (2.4%) and plane travel (4.5%). The prevalence rates of activities performed by individual women did not differ significantly to prevalence rates calculated from all surveys (see Table 2).

**Table 2:** The weekly prevalence rate, per 100 surveys, of activities/events reported in surveys

	Prevalence (95% CI) on all surveys completed	Prevalence (95% CI) on women who completed survey once
Consumed caffeinated drinks	82.6 (79.9 - 85.4)	82.1 (78.9 - 85.2)
Eaten hot and spicy food	47.2 (43.5 - 50.8)	45.3 (41.2 - 49.4)
Had sexual intercourse	42.1 (38.5 - 45.6)	43.1 (39.1 - 47.2)
Lifted anything of more than 12 kilograms	32.7 (29.3 - 36.1)	32.2 (28.3 - 36.0)
Consumed alcohol	21.4 (18.4 - 24.4)	20.6 (17.3 - 23.9)
Performed vigorous physical exercise	6.4 (4.6 - 8.1)	6.6 (4.6 - 8.6)
Had an internal/vaginal examination	6.1 (4.4 - 7.8)	5.7 (3.8 - 7.6)
Onset of an infection	5.0 (3.4 - 6.6)	4.7 (3.1 - 6.6)
Travelled in an aeroplane	4.5 (3.0 - 6.0)	4.5 (2.8 - 6.2)
Taken medication for depression	2.4 (1.3 - 3.6)	2.6 (1.3 - 3.9)

### Prevalence of activities stratified by gestational age

The prevalence rates of most activities did not vary significantly with gestation. However, performing vigorous exercise in the seven days prior to the survey was most prevalent amongst women 29 to 34 weeks pregnant, 11.1% compared to 5.8% and 4.3% for women 20 to 28 weeks pregnant and those over 34 weeks pregnant respectively (p = 0.01). The prevalence of an internal examination increased with gestational age (3.3%, 5.2%, 8.6%; p = 0.03) while the prevalence of plane travel decreased with gestation (6.6%, 5.2%, 2.5%; p = 0.05).

### Prevalence of activities stratified by parity

Compared to women who were pregnant for a second or subsequent time, fewer women who were pregnant for the first time consumed caffeinated drinks (76.9% vs 88.6%, p < 0.001), or lifted something over 12 kilograms (8.2% vs 58.1%, p < 0.001) in the past seven days, while more women having their first baby had had an internal examination (8.2% vs 3.9%, p = 0.01). Although not a statistically significant difference (p = 0.06), more multiparous women had consumed alcohol in the seven days prior to completing the survey than women pregnant for the first time (24.3% vs 18.6%).

### Prevalence of activities stratified by maternal age

The prevalence of lifting something over 12 kilograms in the week prior to survey completion increased with maternal age (p < 0.001) while the prevalence of an internal/vaginal examination decreased with age (p = 0.01). However when these analyses were stratified by parity, there was no longer a significant difference between age groups. Compared to women aged 25 years and older, the prevalence rate for taking medication for depression was higher for women aged less than 25 years (7.3% vs 1.9%, p = 0.01). This difference remained for multiparous women after stratified analysis by parity.

### Prevalence of activities stratified by maternal education

The prevalence of two activities differed significantly by maternal education. Women who had a University degree had higher prevalence rates of lifting something 12 kilograms or more (p = 0.004), although when stratified for parity this difference only remained for multiparous women. Women without University degrees had a higher prevalence of taking medication for depression (4.8% vs 0.7%, p > 0.001), this was significant for both primiparous and multiparous women.

## Discussion

This is the first study to look at the prevalence of activities or events in a seven day window in pregnant women. However there are a number of studies that report the prevalence of select activities during specific trimesters[3,9,11,12] or at any time during pregnancy[3,14,15]. Activities that may be performed regularly during pregnancy and have rates that do not differ with gestational age can be compared to other rates calculated during the whole pregnancy. Prevalence rates similar to our study were recorded for performing vigorous exercise (8% in second trimester)[9], and having sexual intercourse (44% and 45%)[4,6]. One study reported a higher rate of physical activity (49% once or more a week) and a lower rate of sexual intercourse (24%) this could be due to the population of nulliparous, younger women surveyed[11].

Reported rates that differed from the findings from this survey included having medication for depression dispensed, 3.8% during second trimester to 4.1% in the third trimester [13]. Our rate (2.4%) may be lower as we asked if depression medication had been taken in the past week. Another explanation for the difference in results may be the study population. The population in our study is much older than the population reported in this study, with only 7.4% of surveyed women under the age of 25 years in our study compared to 15%. We found that the prevalence of taking medication for depression was significantly higher (7.3%) for women aged less than 25 years. Reported prevalence rates for coffee consumption during pregnancy range from 43%[6] to 57%[16] however the higher prevalence rate of 83% in our study included the consumption of tea and cola drinks. Our finding of prevalence rates of 21% for alcohol consumption during pregnancy were lower than findings from Denmark (44.7%) [17] and Canada (31.2%) [16]. Although we did not ask about quantities of alcohol consumed we believe the majority of women drinking alcohol have only consumed small quantities as in the Canadian study 18% of pregnant women had consumed only one or two drinks per week[16]. Interestingly, at the end of 2007, prior to this study commencing, the National Health and Medical Research Council in Australia publicly released a guideline advising women that it is safest to abstain from alcohol consumption whilst pregnant[18].

For activities that occur less frequently during pregnancy it is necessary to estimate weekly rates for comparison to studies that have collected data over varying lengths of time during pregnancy. Another study found a weekly rate of plane travel for women at least 20 weeks pregnant of approximately 3.3%[6]. Although similar to our finding of 4.5% (3.0% - 6.0%), the population of women attending antenatal clinics at this hospital is of average to advantaged socioeconomic status and therefore may travel by air more than the general population.

We found that for women that had previously been pregnant, the prevalence of lifting something 12 kilograms or more was higher. Infants aged 12 months or more may weigh 12 kilograms[19] and this may also explain the different prevalence rates of heavy lifting between different maternal age groups and education levels. Older women are more likely to have been pregnant before and are also more likely to have a University degree.

A limitation of this study is the use of self reported data which may be affected by recall bias. Mothers' reports of events during pregnancy and labour are generally good[20,21], and are more reliable if less time has elapsed from the event of interest[22]. The seven day period of recall in our study is short and should minimise recall bias. A further limitation of this study is that the study population is older than the general population of pregnant women in Australia[23], is highly educated and speaks English impacting the generalisability of the results, especially for prevalence of activities that differ with maternal age, education or socioeconomic status. Women who completed the survey more than once were more likely to have more complicated pregnancies and increased gestational age as these women attended the antenatal clinic more often.

## Conclusions

This survey is the first study to estimate the weekly prevalence of a number of activities and events in a population of pregnant women. Further research is necessary to investigate whether any of these activities are acute triggers of pregnancy complications. The results of this study can be used to inform such future research or be a guide to clinicians on the prevalence of possibly risky activities amongst different groups of pregnant women.

## Competing interests

The authors declare that they have no competing interests.

## Authors' contributions

SJL designed and administered the survey, analysed data and drafted manuscript. CLR and JBF developed the study and helped design the survey. CLR and RMH contributed to data analysis and interpretation. All authors edited the manuscript and gave final approval of the version to be published.

## Pre-publication history

The pre-publication history for this paper can be accessed here:

http://www.biomedcentral.com/1471-2393/10/41/prepub
